# *trans*-{1,8-Bis[(*S*)-1-phenyl­eth­yl]-1,3,6,8,10,13-hexa­aza­cyclo­tetra­deca­ne}bis(thio­cyanato­-κ*N*)copper(II)

**DOI:** 10.1107/S1600536810026632

**Published:** 2010-07-10

**Authors:** Jong Won Shin, Sankara Rao Rowthu, Jae Jeong Ryoo, Kil Sik Min

**Affiliations:** a Department of Chemistry, Kyungpook National University, Daegu 702-701, Republic of Korea; b Department of Chemistry Education, Kyungpook National University, Daegu 702-701, Republic of Korea

## Abstract

In the title thio­cyanate-coordinated aza-macrocyclic copper(II) complex, [Cu(NCS)_2_(C_24_H_38_N_6_)], the Cu^II^ atom is coordinated by the four secondary N atoms of the aza-macrocyclic ligand and by the two N atoms of the thio­cyanate ions in a tetra­gonally distorted octa­hedral geometry. The average equatorial Cu—N bond length is shorter than the average axial Cu—N bond length [2.010 (2) and 2.528 (4) Å, respectively]. An N—H⋯N hydrogen-bonding inter­action between the secondary amine N atom and the adjacent thio­cyanate ion leads to a polymeric chain along the *a* axis.

## Related literature

For the potential applications of chiral metal complexes in chiral recognition and chiral catalysis, see: Katsuki *et al.* (2000 ▶); Lehn (1995 ▶) and as chiral building blocks, see: Du *et al.* (2003 ▶); Gao *et al.* (2005 ▶). It has been reported that the enanti­omers of [Ru(1,10-phenanthroline)_3_]^2+^ induce chiral aggregation of various achiral anionic porphyrins, see: Randazzo *et al.* (2008 ▶). For typical C—S bond lengths, see: Banerjee & Zubieta (2004 ▶); Stølevik & Postmyr (1997 ▶). For the preparation, see: Han *et al.* (2008 ▶).
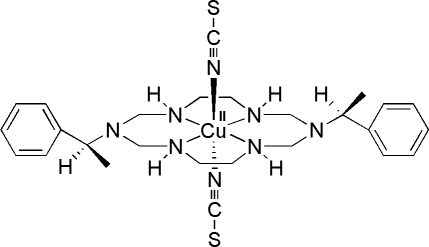


## Experimental

### 

#### Crystal data

[Cu(NCS)_2_(C_24_H_38_N_6_)]
*M* *_r_* = 590.30
Monoclinic, 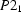

*a* = 6.5976 (5) Å
*b* = 14.7609 (11) Å
*c* = 15.2847 (12) Å
β = 99.952 (2)°
*V* = 1466.13 (19) Å^3^
*Z* = 2
Mo *K*α radiation
μ = 0.92 mm^−1^
*T* = 195 K
0.38 × 0.26 × 0.15 mm

#### Data collection

Siemens SMART CCD diffractometer
Absorption correction: multi-scan (*SADABS*; Sheldrick, 1996 ▶) *T*_min_ = 0.751, *T*_max_ = 0.871
10954 measured reflections
6272 independent reflections
4364 reflections with *I* > 2σ(*I*)
*R*_int_ = 0.034

#### Refinement

*R*[*F* ^2^ > 2σ(*F* ^2^)] = 0.048
*wR*(*F* ^2^) = 0.115
*S* = 1.11
6272 reflections
336 parameters
1 restraint
H-atom parameters constrained
Δρ_max_ = 0.69 e Å^−3^
Δρ_min_ = −0.68 e Å^−3^
Absolute structure: Flack (1983 ▶), 2485 Friedel pairs
Flack parameter: −0.01 (2)

### 

Data collection: *SMART* (Siemens, 1996 ▶); cell refinement: *SAINT* (Siemens, 1996 ▶); data reduction: *SHELXTL* (Sheldrick, 2008 ▶); program(s) used to solve structure: *SHELXS97* (Sheldrick, 2008 ▶); program(s) used to refine structure: *SHELXL97* (Sheldrick, 2008 ▶); molecular graphics: *ORTEP-3* (Farrugia, 1997 ▶); software used to prepare material for publication: *SHELXL97*.

## Supplementary Material

Crystal structure: contains datablocks global, I. DOI: 10.1107/S1600536810026632/jh2175sup1.cif

Structure factors: contains datablocks I. DOI: 10.1107/S1600536810026632/jh2175Isup2.hkl

Additional supplementary materials:  crystallographic information; 3D view; checkCIF report

## Figures and Tables

**Table 1:** Hydrogen-bond geometry (Å, °)

*D*—H⋯*A*	*D*—H	H⋯*A*	*D*⋯*A*	*D*—H⋯*A*
N1—H1⋯N7^i^	0.93	2.54	3.258 (7)	135
N4—H4⋯N8^ii^	0.93	2.46	3.202 (7)	137

Symmetry codes: (i) 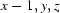; (ii) 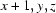.

**Fig. 1.**
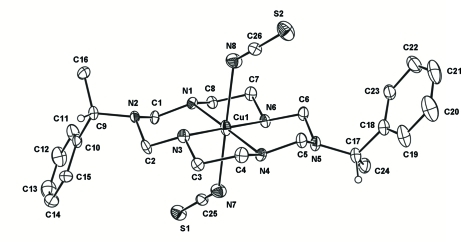
ORTEP drawing of the molecular title compound with atomic numbering scheme at 30% probability.

**Fig. 2.**
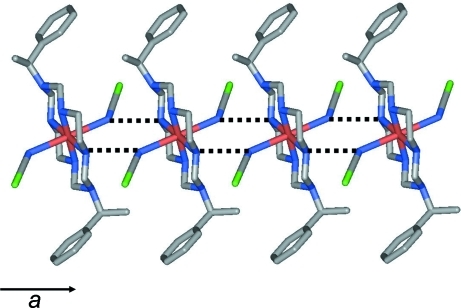
Perspective view of the title compound showing a one-dimensional chain formed by N—H···N (of NCS) hydrogen bonding interactions.

## supplementary crystallographic information

### Comment

Chiral metal complexes have attracted considerable attention in chemistry and material science because of their potential applications for chiral recognition and chiral catalysis (Lehn, 1995; Katsuki *et al.*, 2000). Very recently, it has reported the enantiomers of [Ru(1,10-phenanthroline)_3_]^2+^ induce chiral aggregation of various achiral anionic porphyrins and that the complexes can transfer molecular information, *i.e.* energy and chirality (Randazzo *et al.*, 2008). However, the study of chiral macrocyclic metal complexes has been limited due to the difficult of preparation, although these complexes are ve ry useful for chiral building blocks (Du *et al.*, 2003; Gao *et al.*, 2005). Here, we report the synthesis and crystal structure of copper(II) azamacrocyclic chiral complex, *trans*-Dithiocyanato(1,8-di(*S*-α-methylbenzyl)- 1,3,6,8,10,13-hexaazacyclotetradecane)copper(II), with two thiocyanate ions axially.

In the title compound, the coordination geometry around copper(II) ion is a tetragonally distorted octahedron in which copper(II) ion is coordinated to the four secondary N atoms of the azamacrocyclic ligand in the square-planar fashion and two N atoms from the thiocyanate ions at the axial positions as shown in Figure 1. The average Ni—N_eq_ and Ni—N_ax_ bond distances are 2.010 (2) and 2.528 (4) Å, respectively. The former is much less than the latter, which can be attributed to a rather large Jahn-Teller distortion of the copper(II) ion and/or the ring contraction of the azamacrocyclic ligand. In the coordinated thiocyanate ions, the average N—C and C—S bond distances are 1.160 (6) and 1.638 (5) Å, respectively. The former is very similar to CN triple bond length, while the latter is slightly shorter than the normal CS single bond distance (Stølevik & Postmyr, 1997; Banerjee & Zubieta, 2004). The pendant arms of azamacrocyclic ligand have chiral carbon atoms (S type). All thiocyanate ions binding copper(II) ions axially are involved in an N—H···N(of NCS) hydrogen bonding interactions (Table 1), which gives rise to a one-dimensional polymeric chain propagating along the *a* axis (Figure 2). The shortest Cu···Cu intrachain separation within the hydrogen-bonded one-dimensional polymer is 6.598 (1) Å and is about 37% shorter than the shortest interchain Cu···Cu distance of 10.448 (1) Å.

### Experimental

The title compound is prepared as follows. [Cu(C_24_H_38_N_6_)](ClO_4_)_2_ was prepared by a slightly modified literature procedure: as CuCl_2_.2H_2_O and *S*-(-)-1-Phenylethylamine were used instead of NiCl_2_.6H_2_O and *R*-(+)-1-Phenylethylamine (Han *et al.*, 2008). To an MeCN solution (10 ml) of [Cu(C_24_H_38_N_6_)](ClO_4_)_2_ (90 mg, 0.15 mmol) was added dropwise an aqueous solution (10 ml) containing NaSCN (24 mg, 0.30 mmol) at ambient temperature. The color of the solution changed to pale pink. The mixture was stirred for 30 min during which time a pink precipitate of formed which was collected by filtration, washed with MeCN and water, and dried in air. Single crystals of the title compound suitable for X-ray crystallography were grown by layering of the MeCN solution of [Cu(C_24_H_38_N_6_)](ClO_4_)_2_ on the aqueous solution of NaSCN within one week.

### Refinement

All H atoms in the title compound were placed in geometrically idealized positions and constrained to ride on their parent atoms, with C—H distances of 0.95 (ring H atoms) or 0.99–1.00 (open chain H atoms) Å and N—H distance of 0.93 Å, and with *U*_iso_(H) values of 1.2 times the equivalent anisotropic displacement parameters of the parent C and N atoms.

### Crystal data

**Table:**

[Cu(NCS)_2_(C_24_H_38_N_6_)]	*F*(000) = 622
*M**_r_* = 590.30	*D*_x_ = 1.337 Mg m^−^^3^
Monoclinic, *P*2_1_	Mo *K*α radiation, λ = 0.71073 Å
Hall symbol: P 2yb	Cell parameters from 4491 reflections
*a* = 6.5976 (5) Å	θ = 2.7–27.9°
*b* = 14.7609 (11) Å	µ = 0.92 mm^−^^1^
*c* = 15.2847 (12) Å	*T* = 195 K
β = 99.952 (2)°	Block, purple
*V* = 1466.13 (19) Å^3^	0.38 × 0.26 × 0.15 mm
*Z* = 2	

### Data collection

**Table:**

Siemens SMART CCD diffractometer	6272 independent reflections
Radiation source: fine-focus sealed tube	4364 reflections with *I* > 2σ(*I*)
graphite	*R*_int_ = 0.034
φ and ω scans	θ_max_ = 28.3°, θ_min_ = 1.4°
Absorption correction: multi-scan (*SADABS*; Sheldrick, 1996)	*h* = −6→8
*T*_min_ = 0.751, *T*_max_ = 0.871	*k* = −19→19
10954 measured reflections	*l* = −20→20

### Refinement

**Table:**

Refinement on *F*^2^	Secondary atom site location: difference Fourier map
Least-squares matrix: full	Hydrogen site location: inferred from neighbouring sites
*R*[*F*^2^ > 2σ(*F*^2^)] = 0.048	H-atom parameters constrained
*wR*(*F*^2^) = 0.115	*w* = 1/[σ^2^(*F*_o_^2^) + (0.0195*P*)^2^ + 1.3207*P*] where *P* = (*F*_o_^2^ + 2*F*_c_^2^)/3
*S* = 1.11	(Δ/σ)_max_ = 0.001
6272 reflections	Δρ_max_ = 0.69 e Å^−^^3^
336 parameters	Δρ_min_ = −0.67 e Å^−^^3^
1 restraint	Absolute structure: Flack (1983), 2485 Friedel pairs
Primary atom site location: structure-invariant direct methods	Flack parameter: −0.01 (2)

### Special details

**Table:**

Geometry. All e.s.d.'s (except the e.s.d. in the dihedral angle between two l.s. planes) are estimated using the full covariance matrix. The cell e.s.d.'s are taken into account individually in the estimation of e.s.d.'s in distances, angles and torsion angles; correlations between e.s.d.'s in cell parameters are only used when they are defined by crystal symmetry. An approximate (isotropic) treatment of cell e.s.d.'s is used for estimating e.s.d.'s involving l.s. planes.
Refinement. Refinement of *F*^2^ against ALL reflections. The weighted *R*-factor *wR* and goodness of fit *S* are based on *F*^2^, conventional *R*-factors *R* are based on *F*, with *F* set to zero for negative *F*^2^. The threshold expression of *F*^2^ > σ(*F*^2^) is used only for calculating *R*-factors(gt) *etc*. and is not relevant to the choice of reflections for refinement. *R*-factors based on *F*^2^ are statistically about twice as large as those based on *F*, and *R*- factors based on ALL data will be even larger.

### Fractional atomic coordinates and isotropic or equivalent isotropic displacement parameters (Å^2^)

**Table:**

	*x*	*y*	*z*	*U*_iso_*/*U*_eq_	
Cu1	0.50430 (12)	0.54334 (8)	0.25843 (4)	0.03442 (15)	
S1	0.7793 (3)	0.26210 (11)	0.15869 (12)	0.0543 (5)	
S2	0.2363 (3)	0.82615 (11)	0.34351 (12)	0.0583 (5)	
N1	0.2789 (7)	0.4510 (3)	0.2305 (2)	0.0254 (9)	
H1	0.1630	0.4766	0.2476	0.030*	
N2	0.3528 (7)	0.3783 (3)	0.3734 (3)	0.0274 (10)	
N3	0.5658 (7)	0.5102 (3)	0.3877 (2)	0.0303 (11)	
H3	0.4650	0.5373	0.4146	0.036*	
N4	0.7316 (7)	0.6346 (3)	0.2878 (2)	0.0273 (10)	
H4	0.8493	0.6078	0.2736	0.033*	
N5	0.6763 (8)	0.7003 (3)	0.1436 (3)	0.0342 (11)	
N6	0.4452 (7)	0.5748 (3)	0.1283 (2)	0.0294 (11)	
H6	0.5388	0.5424	0.1015	0.035*	
N7	0.7810 (9)	0.4420 (4)	0.2165 (3)	0.0486 (14)	
N8	0.2189 (8)	0.6434 (4)	0.2945 (3)	0.0484 (14)	
C1	0.3104 (9)	0.3626 (4)	0.2767 (3)	0.0297 (12)	
H1A	0.1858	0.3246	0.2609	0.036*	
H1B	0.4275	0.3303	0.2582	0.036*	
C2	0.5574 (9)	0.4111 (4)	0.4052 (3)	0.0339 (13)	
H2A	0.6567	0.3789	0.3746	0.041*	
H2B	0.5953	0.3994	0.4697	0.041*	
C3	0.7627 (7)	0.5530 (5)	0.4258 (3)	0.0288 (12)	
H3A	0.8787	0.5158	0.4127	0.035*	
H3B	0.7747	0.5582	0.4910	0.035*	
C4	0.7689 (9)	0.6463 (4)	0.3847 (3)	0.0359 (14)	
H4A	0.6618	0.6857	0.4027	0.043*	
H4B	0.9049	0.6748	0.4047	0.043*	
C5	0.7015 (10)	0.7205 (4)	0.2374 (3)	0.0341 (13)	
H5A	0.5780	0.7522	0.2505	0.041*	
H5B	0.8222	0.7605	0.2549	0.041*	
C6	0.4694 (9)	0.6717 (4)	0.1056 (3)	0.0345 (13)	
H6A	0.4461	0.6794	0.0403	0.041*	
H6B	0.3674	0.7091	0.1298	0.041*	
C7	0.2393 (8)	0.5371 (5)	0.0934 (3)	0.0348 (12)	
H7A	0.2174	0.5339	0.0278	0.042*	
H7B	0.1307	0.5758	0.1111	0.042*	
C8	0.2317 (9)	0.4425 (4)	0.1326 (3)	0.0311 (12)	
H8A	0.0933	0.4158	0.1142	0.037*	
H8B	0.3339	0.4027	0.1115	0.037*	
C9	0.2883 (9)	0.3009 (4)	0.4249 (3)	0.0361 (13)	
H9	0.3499	0.3132	0.4883	0.043*	
C10	0.3729 (9)	0.2099 (3)	0.4034 (3)	0.0331 (13)	
C11	0.2610 (11)	0.1501 (5)	0.3414 (4)	0.0534 (18)	
H11	0.1285	0.1674	0.3112	0.064*	
C12	0.3396 (14)	0.0674 (5)	0.3239 (4)	0.066 (2)	
H12	0.2606	0.0278	0.2824	0.079*	
C13	0.5321 (13)	0.0415 (7)	0.3659 (4)	0.0688 (19)	
H13	0.5858	−0.0158	0.3530	0.083*	
C14	0.6468 (11)	0.0979 (5)	0.4265 (4)	0.0541 (18)	
H14	0.7794	0.0797	0.4558	0.065*	
C15	0.5689 (9)	0.1810 (4)	0.4445 (4)	0.0402 (14)	
H15	0.6501	0.2199	0.4860	0.048*	
C16	0.0579 (8)	0.3032 (4)	0.4214 (4)	0.0473 (15)	
H16A	−0.0116	0.3002	0.3594	0.071*	
H16B	0.0197	0.3595	0.4483	0.071*	
H16C	0.0163	0.2513	0.4542	0.071*	
C17	0.7789 (9)	0.7675 (4)	0.0932 (3)	0.0380 (14)	
H17	0.9250	0.7709	0.1244	0.046*	
C18	0.6930 (9)	0.8627 (4)	0.0969 (3)	0.0390 (14)	
C19	0.8066 (12)	0.9284 (5)	0.1483 (4)	0.059 (2)	
H19	0.9418	0.9143	0.1778	0.071*	
C20	0.7286 (15)	1.0139 (5)	0.1579 (4)	0.074 (3)	
H20	0.8100	1.0583	0.1928	0.089*	
C21	0.5318 (14)	1.0343 (6)	0.1165 (4)	0.070 (2)	
H21	0.4761	1.0925	0.1243	0.084*	
C22	0.4137 (12)	0.9710 (5)	0.0634 (4)	0.060 (2)	
H22	0.2793	0.9855	0.0334	0.072*	
C23	0.4972 (10)	0.8857 (4)	0.0553 (4)	0.0453 (16)	
H23	0.4166	0.8415	0.0198	0.054*	
C24	0.7881 (9)	0.7340 (4)	0.0004 (3)	0.0456 (14)	
H24A	0.8273	0.6699	0.0028	0.068*	
H24B	0.8902	0.7693	−0.0246	0.068*	
H24C	0.6527	0.7411	−0.0371	0.068*	
C25	0.7772 (9)	0.3680 (5)	0.1908 (4)	0.0360 (14)	
C26	0.2274 (9)	0.7192 (4)	0.3148 (4)	0.0368 (14)	

### Atomic displacement parameters (Å^2^)

**Table:**

	*U*^11^	*U*^22^	*U*^33^	*U*^12^	*U*^13^	*U*^23^
Cu1	0.0461 (3)	0.0263 (2)	0.0280 (3)	−0.0102 (3)	−0.0019 (2)	0.0032 (3)
S1	0.0567 (12)	0.0369 (10)	0.0655 (11)	−0.0006 (9)	−0.0005 (9)	−0.0091 (8)
S2	0.0683 (13)	0.0330 (10)	0.0674 (11)	−0.0024 (9)	−0.0058 (9)	−0.0068 (8)
N1	0.029 (2)	0.020 (2)	0.028 (2)	−0.0074 (19)	0.0044 (17)	−0.0053 (16)
N2	0.027 (2)	0.024 (2)	0.031 (2)	−0.0027 (19)	0.0019 (18)	0.0087 (17)
N3	0.035 (3)	0.027 (2)	0.029 (2)	−0.004 (2)	0.0039 (18)	−0.0033 (16)
N4	0.029 (2)	0.023 (2)	0.029 (2)	−0.008 (2)	0.0031 (17)	−0.0032 (18)
N5	0.041 (3)	0.033 (3)	0.028 (2)	−0.008 (2)	0.004 (2)	0.0076 (19)
N6	0.040 (3)	0.023 (2)	0.0237 (19)	−0.003 (2)	0.0022 (18)	−0.0013 (15)
N7	0.048 (4)	0.036 (3)	0.062 (3)	0.007 (3)	0.012 (3)	−0.007 (3)
N8	0.047 (4)	0.034 (3)	0.063 (3)	0.001 (3)	0.009 (3)	−0.009 (3)
C1	0.039 (3)	0.020 (3)	0.031 (3)	−0.008 (2)	0.008 (2)	0.002 (2)
C2	0.041 (3)	0.023 (3)	0.033 (3)	0.003 (3)	−0.005 (2)	0.011 (2)
C3	0.031 (3)	0.028 (3)	0.026 (2)	−0.001 (3)	−0.0013 (18)	−0.003 (2)
C4	0.043 (4)	0.039 (3)	0.024 (2)	−0.004 (3)	0.002 (2)	−0.002 (2)
C5	0.042 (4)	0.025 (3)	0.033 (3)	−0.006 (3)	0.002 (2)	0.004 (2)
C6	0.039 (3)	0.033 (3)	0.031 (3)	−0.006 (3)	0.004 (2)	0.008 (2)
C7	0.041 (3)	0.039 (3)	0.0217 (19)	−0.007 (3)	−0.0029 (18)	−0.001 (3)
C8	0.040 (3)	0.026 (3)	0.027 (2)	0.001 (3)	0.006 (2)	−0.001 (2)
C9	0.041 (3)	0.036 (3)	0.033 (3)	0.002 (3)	0.010 (2)	0.015 (2)
C10	0.041 (3)	0.021 (3)	0.038 (3)	−0.004 (2)	0.009 (2)	0.008 (2)
C11	0.064 (5)	0.040 (4)	0.050 (4)	−0.008 (3)	−0.006 (3)	0.011 (3)
C12	0.097 (6)	0.041 (4)	0.056 (4)	−0.009 (4)	0.005 (4)	−0.005 (3)
C13	0.099 (6)	0.045 (4)	0.068 (4)	0.008 (5)	0.029 (4)	0.000 (5)
C14	0.051 (4)	0.043 (4)	0.073 (4)	0.014 (3)	0.023 (3)	0.011 (3)
C15	0.029 (3)	0.041 (3)	0.050 (3)	0.003 (3)	0.007 (3)	0.007 (3)
C16	0.027 (3)	0.059 (4)	0.056 (4)	−0.003 (3)	0.009 (3)	0.021 (3)
C17	0.050 (4)	0.025 (3)	0.040 (3)	−0.003 (3)	0.010 (3)	0.005 (2)
C18	0.050 (4)	0.032 (3)	0.035 (3)	−0.003 (3)	0.007 (3)	0.008 (2)
C19	0.091 (6)	0.031 (3)	0.048 (4)	−0.009 (4)	−0.007 (4)	0.007 (3)
C20	0.123 (8)	0.042 (4)	0.050 (4)	−0.005 (4)	−0.009 (4)	0.001 (3)
C21	0.131 (7)	0.026 (3)	0.058 (4)	0.002 (5)	0.029 (4)	0.000 (4)
C22	0.077 (5)	0.046 (4)	0.059 (4)	0.021 (4)	0.021 (4)	0.018 (3)
C23	0.056 (4)	0.032 (3)	0.051 (3)	0.004 (3)	0.018 (3)	0.009 (3)
C24	0.058 (4)	0.040 (3)	0.044 (3)	0.002 (3)	0.022 (3)	0.007 (3)
C25	0.032 (3)	0.042 (4)	0.033 (3)	0.004 (3)	0.003 (2)	0.005 (3)
C26	0.033 (3)	0.037 (4)	0.040 (3)	0.005 (3)	0.005 (3)	0.004 (3)

### Geometric parameters (Å, °)

**Table:**

Cu1—N3	2.008 (4)	C6—H6B	0.9900
Cu1—N4	2.008 (4)	C7—C8	1.523 (9)
Cu1—N1	2.008 (4)	C7—H7A	0.9900
Cu1—N6	2.014 (4)	C7—H7B	0.9900
Cu1—N7	2.527 (6)	C8—H8A	0.9900
Cu1—N8	2.528 (6)	C8—H8B	0.9900
S1—C25	1.639 (7)	C9—C16	1.512 (7)
S2—C26	1.636 (7)	C9—C10	1.513 (8)
N1—C8	1.480 (6)	C9—H9	1.0000
N1—C1	1.482 (6)	C10—C15	1.403 (7)
N1—H1	0.9300	C10—C11	1.406 (8)
N2—C2	1.437 (7)	C11—C12	1.372 (10)
N2—C1	1.474 (6)	C11—H11	0.9500
N2—C9	1.490 (6)	C12—C13	1.375 (10)
N3—C3	1.470 (7)	C12—H12	0.9500
N3—C2	1.490 (6)	C13—C14	1.372 (10)
N3—H3	0.9300	C13—H13	0.9500
N4—C4	1.469 (6)	C14—C15	1.376 (8)
N4—C5	1.479 (7)	C14—H14	0.9500
N4—H4	0.9300	C15—H15	0.9500
N5—C5	1.445 (6)	C16—H16A	0.9800
N5—C6	1.450 (7)	C16—H16B	0.9800
N5—C17	1.490 (7)	C16—H16C	0.9800
N6—C7	1.480 (7)	C17—C24	1.514 (7)
N6—C6	1.487 (7)	C17—C18	1.520 (8)
N6—H6	0.9300	C17—H17	1.0000
N7—C25	1.160 (8)	C18—C23	1.379 (8)
N8—C26	1.160 (8)	C18—C19	1.384 (9)
C1—H1A	0.9900	C19—C20	1.381 (10)
C1—H1B	0.9900	C19—H19	0.9500
C2—H2A	0.9900	C20—C21	1.376 (11)
C2—H2B	0.9900	C20—H20	0.9500
C3—C4	1.518 (8)	C21—C22	1.385 (11)
C3—H3A	0.9900	C21—H21	0.9500
C3—H3B	0.9900	C22—C23	1.389 (9)
C4—H4A	0.9900	C22—H22	0.9500
C4—H4B	0.9900	C23—H23	0.9500
C5—H5A	0.9900	C24—H24A	0.9800
C5—H5B	0.9900	C24—H24B	0.9800
C6—H6A	0.9900	C24—H24C	0.9800
			
N3—Cu1—N4	85.80 (17)	N6—C7—H7A	110.3
N3—Cu1—N1	93.45 (17)	C8—C7—H7A	110.3
N4—Cu1—N1	179.2 (2)	N6—C7—H7B	110.3
N3—Cu1—N6	179.1 (2)	C8—C7—H7B	110.3
N4—Cu1—N6	94.36 (17)	H7A—C7—H7B	108.6
N1—Cu1—N6	86.38 (17)	N1—C8—C7	107.8 (4)
C8—N1—C1	113.3 (4)	N1—C8—H8A	110.2
C8—N1—Cu1	106.9 (3)	C7—C8—H8A	110.2
C1—N1—Cu1	117.3 (3)	N1—C8—H8B	110.2
C8—N1—H1	106.2	C7—C8—H8B	110.2
C1—N1—H1	106.2	H8A—C8—H8B	108.5
Cu1—N1—H1	106.2	N2—C9—C16	110.0 (4)
C2—N2—C1	113.3 (4)	N2—C9—C10	114.6 (4)
C2—N2—C9	114.7 (4)	C16—C9—C10	114.7 (5)
C1—N2—C9	112.7 (4)	N2—C9—H9	105.6
C3—N3—C2	114.1 (4)	C16—C9—H9	105.6
C3—N3—Cu1	107.4 (3)	C10—C9—H9	105.6
C2—N3—Cu1	114.1 (3)	C15—C10—C11	116.6 (6)
C3—N3—H3	106.9	C15—C10—C9	121.2 (5)
C2—N3—H3	106.9	C11—C10—C9	122.2 (5)
Cu1—N3—H3	106.9	C12—C11—C10	121.2 (7)
C4—N4—C5	114.1 (4)	C12—C11—H11	119.4
C4—N4—Cu1	107.1 (3)	C10—C11—H11	119.4
C5—N4—Cu1	115.5 (3)	C11—C12—C13	120.5 (7)
C4—N4—H4	106.5	C11—C12—H12	119.8
C5—N4—H4	106.5	C13—C12—H12	119.8
Cu1—N4—H4	106.5	C14—C13—C12	120.2 (8)
C5—N5—C6	113.4 (5)	C14—C13—H13	119.9
C5—N5—C17	113.0 (4)	C12—C13—H13	119.9
C6—N5—C17	117.8 (4)	C13—C14—C15	119.7 (7)
C7—N6—C6	114.0 (5)	C13—C14—H14	120.2
C7—N6—Cu1	106.2 (3)	C15—C14—H14	120.2
C6—N6—Cu1	116.2 (3)	C14—C15—C10	121.9 (6)
C7—N6—H6	106.6	C14—C15—H15	119.0
C6—N6—H6	106.6	C10—C15—H15	119.0
Cu1—N6—H6	106.6	C9—C16—H16A	109.5
N2—C1—N1	109.0 (4)	C9—C16—H16B	109.5
N2—C1—H1A	109.9	H16A—C16—H16B	109.5
N1—C1—H1A	109.9	C9—C16—H16C	109.5
N2—C1—H1B	109.9	H16A—C16—H16C	109.5
N1—C1—H1B	109.9	H16B—C16—H16C	109.5
H1A—C1—H1B	108.3	N5—C17—C24	111.2 (4)
N2—C2—N3	109.4 (4)	N5—C17—C18	113.0 (5)
N2—C2—H2A	109.8	C24—C17—C18	114.4 (4)
N3—C2—H2A	109.8	N5—C17—H17	105.8
N2—C2—H2B	109.8	C24—C17—H17	105.8
N3—C2—H2B	109.8	C18—C17—H17	105.8
H2A—C2—H2B	108.2	C23—C18—C19	117.5 (6)
N3—C3—C4	108.1 (4)	C23—C18—C17	122.4 (6)
N3—C3—H3A	110.1	C19—C18—C17	120.0 (6)
C4—C3—H3A	110.1	C20—C19—C18	121.7 (7)
N3—C3—H3B	110.1	C20—C19—H19	119.2
C4—C3—H3B	110.1	C18—C19—H19	119.2
H3A—C3—H3B	108.4	C21—C20—C19	119.4 (7)
N4—C4—C3	107.3 (4)	C21—C20—H20	120.3
N4—C4—H4A	110.2	C19—C20—H20	120.3
C3—C4—H4A	110.2	C20—C21—C22	120.8 (7)
N4—C4—H4B	110.2	C20—C21—H21	119.6
C3—C4—H4B	110.2	C22—C21—H21	119.6
H4A—C4—H4B	108.5	C21—C22—C23	118.2 (7)
N5—C5—N4	108.8 (4)	C21—C22—H22	120.9
N5—C5—H5A	109.9	C23—C22—H22	120.9
N4—C5—H5A	109.9	C18—C23—C22	122.4 (7)
N5—C5—H5B	109.9	C18—C23—H23	118.8
N4—C5—H5B	109.9	C22—C23—H23	118.8
H5A—C5—H5B	108.3	C17—C24—H24A	109.5
N5—C6—N6	108.5 (4)	C17—C24—H24B	109.5
N5—C6—H6A	110.0	H24A—C24—H24B	109.5
N6—C6—H6A	110.0	C17—C24—H24C	109.5
N5—C6—H6B	110.0	H24A—C24—H24C	109.5
N6—C6—H6B	110.0	H24B—C24—H24C	109.5
H6A—C6—H6B	108.4	N7—C25—S1	177.3 (6)
N6—C7—C8	107.1 (4)	N8—C26—S2	179.3 (6)
			
N3—Cu1—N1—C8	−166.4 (3)	C6—N6—C7—C8	−172.6 (4)
N6—Cu1—N1—C8	12.7 (3)	Cu1—N6—C7—C8	−43.4 (5)
N3—Cu1—N1—C1	−38.0 (4)	C1—N1—C8—C7	−170.5 (4)
N6—Cu1—N1—C1	141.1 (4)	Cu1—N1—C8—C7	−39.7 (5)
N4—Cu1—N3—C3	−12.5 (4)	N6—C7—C8—N1	56.3 (6)
N1—Cu1—N3—C3	167.1 (4)	C2—N2—C9—C16	150.3 (5)
N4—Cu1—N3—C2	−140.0 (4)	C1—N2—C9—C16	−78.0 (6)
N1—Cu1—N3—C2	39.6 (4)	C2—N2—C9—C10	−78.8 (6)
N3—Cu1—N4—C4	−16.9 (4)	C1—N2—C9—C10	52.9 (6)
N6—Cu1—N4—C4	164.0 (4)	N2—C9—C10—C15	84.8 (6)
N3—Cu1—N4—C5	−145.2 (4)	C16—C9—C10—C15	−146.6 (5)
N6—Cu1—N4—C5	35.7 (4)	N2—C9—C10—C11	−95.1 (6)
N4—Cu1—N6—C7	−162.9 (4)	C16—C9—C10—C11	33.5 (7)
N1—Cu1—N6—C7	17.4 (4)	C15—C10—C11—C12	1.1 (9)
N4—Cu1—N6—C6	−35.0 (4)	C9—C10—C11—C12	−179.1 (6)
N1—Cu1—N6—C6	145.4 (4)	C10—C11—C12—C13	−0.9 (11)
C2—N2—C1—N1	−75.4 (6)	C11—C12—C13—C14	0.5 (11)
C9—N2—C1—N1	152.3 (4)	C12—C13—C14—C15	−0.5 (11)
C8—N1—C1—N2	−179.1 (4)	C13—C14—C15—C10	0.7 (10)
Cu1—N1—C1—N2	55.6 (5)	C11—C10—C15—C14	−1.0 (9)
C1—N2—C2—N3	80.1 (5)	C9—C10—C15—C14	179.1 (5)
C9—N2—C2—N3	−148.6 (4)	C5—N5—C17—C24	168.0 (5)
C3—N3—C2—N2	174.0 (4)	C6—N5—C17—C24	−56.7 (7)
Cu1—N3—C2—N2	−62.0 (5)	C5—N5—C17—C18	−61.8 (6)
C2—N3—C3—C4	166.4 (4)	C6—N5—C17—C18	73.6 (6)
Cu1—N3—C3—C4	38.9 (5)	N5—C17—C18—C23	−68.8 (7)
C5—N4—C4—C3	171.2 (4)	C24—C17—C18—C23	59.8 (7)
Cu1—N4—C4—C3	42.1 (5)	N5—C17—C18—C19	106.6 (6)
N3—C3—C4—N4	−54.6 (6)	C24—C17—C18—C19	−124.8 (6)
C6—N5—C5—N4	81.0 (6)	C23—C18—C19—C20	−0.1 (10)
C17—N5—C5—N4	−141.6 (5)	C17—C18—C19—C20	−175.8 (6)
C4—N4—C5—N5	177.3 (5)	C18—C19—C20—C21	1.0 (11)
Cu1—N4—C5—N5	−58.0 (6)	C19—C20—C21—C22	−1.8 (11)
C5—N5—C6—N6	−79.4 (5)	C20—C21—C22—C23	1.8 (10)
C17—N5—C6—N6	145.4 (5)	C19—C18—C23—C22	0.2 (9)
C7—N6—C6—N5	179.9 (4)	C17—C18—C23—C22	175.7 (5)
Cu1—N6—C6—N5	55.9 (6)	C21—C22—C23—C18	−1.0 (9)

### Hydrogen-bond geometry (Å, °)

**Table:**

*D*—H···*A*	*D*—H	H···*A*	*D*···*A*	*D*—H···*A*
N1—H1···N7^i^	0.93	2.54	3.258 (7)	135
N4—H4···N8^ii^	0.93	2.46	3.202 (7)	137

Symmetry codes: (i) *x*−1, *y*, *z*; (ii) *x*+1, *y*, *z*.

## References

[bb1] Banerjee, S. R. & Zubieta, J. (2004). *Acta Cryst.* C**60**, m208–m209. 10.1107/S0108270104006869 15131366

[bb2] Du, G., Ellern, A. & Woo, L. K. (2003). *Inorg. Chem.* **42**, 873–877. 10.1021/ic025818v 12562202

[bb3] Farrugia, L. J. (1997). *J. Appl. Cryst.* **30**, 565.

[bb13] Flack, H. D. (1983). *Acta Cryst.* A**39**, 876–881.

[bb4] Gao, J., Reibenspies, J. H., Zingaro, R. A., Woolley, F. R., Martell, A. E. & Clearfield, A. (2005). *Inorg. Chem.* **44**, 232–241. 10.1021/ic049181m 15651868

[bb5] Han, J. H., Cha, M. J., Kim, B. G., Kim, S. K. & Min, K. S. (2008). *Inorg. Chem. Commun.* **11**, 745–748.

[bb6] Katsuki, I., Matsumoto, N. & Kojima, M. (2000). *Inorg. Chem.* **39**, 3350–3354. 10.1021/ic9909025 11196874

[bb7] Lehn, J.-M. (1995). *Supramolecular Chemistry*; *Concepts and Perspectives* Weinheim: VCH.

[bb8] Randazzo, R., Mammana, A., D’Urso, A., Lauceri, R. & Purrello, R. (2008). *Angew. Chem. Int. Ed.* **47**, 9879–9882. 10.1002/anie.200803619 19016279

[bb9] Sheldrick, G. M. (1996). *SADABS* University of Göttingen, Germany.

[bb10] Sheldrick, G. M. (2008). *Acta Cryst.* A**64**, 112–122. 10.1107/S0108767307043930 18156677

[bb11] Siemens (1996). *SMART *and *SAINT* Siemens Analytical X-ray Instruments Inc., Madison, Wisconsin, USA.

[bb12] Stølevik, R. & Postmyr, L. (1997). *J. Mol. Struct.* **403**, 207–211.

